# Efficacy of P62-expressing plasmid in treatment of canine osteoarthritis pain: a pilot study

**DOI:** 10.3389/fvets.2025.1519881

**Published:** 2025-06-06

**Authors:** Vladimir Gabai, Evgeny Bakin, Maxim Langs, Robert Devlin, Sergei Krasny, Yauheni Baranou, Sergey Polyakov, Maksim Patapovich, Sergey Gvozdev, Maksim Kardash, Aliaksei Bazyleuski, Andrei Yeliseyeu, Egor Lelikov, Andrei Borodko, Alexander Shneider

**Affiliations:** ^1^CureLab Veterinary Inc., Boston, MA, United States; ^2^CytoReason LTD, Tel-Aviv, Israel; ^3^Miramonte High School, Orinda, CA, United States; ^4^N.N. Alexandrov National Cancer Centre of Belarus, Minsk, Belarus; ^5^Minsk City Clinical Oncologic Centre, Minsk, Belarus; ^6^Department of Microbiology, Faculty of Biology, Belarusian State University, Minsk, Belarus; ^7^BELVITUNIFARM, Vitebsk, Belarus; ^8^Doctor Vet Veterinary Сlinic, Minsk, Belarus; ^9^Dr. A. Bazylevsky Veterinary Center, Vitebsk, Belarus; ^10^Veterinary Clinic AZBUKAVET, Grodno, Belarus; ^11^PoliVetClinic, Gomel, Belarus; ^12^Alpha-Vet Private Unitary Enterprise, Minsk, Belarus; ^13^Department of Molecular Biology, Ariel University, Ariel, Israel

**Keywords:** DNA plasmid, inflammation, chronic pain, pilot trial, safety

## Abstract

**Introduction:**

Osteoarthritis (OA) is a progressive degenerative disease of synovial joints which is highly prevalent in dogs and results in lameness, loss of joint function and mobility, chronic pain, and reduced quality of life. Traditional OA management consist of non-steroidal anti-inflammatory drugs and remains challenging because of significant side effects, thus there is an urgent need for new effective and safe therapeutics for OA.

**Methods:**

Here we present the results of our one-arm open-label pilot clinical study of our novel biologics, a DNA plasmid encoding SQSTM/p62, in 17 companion dogs suffering from OA. The dogs were injected intramuscularly with p62-plasmid once a week for 10 weeks, and pain relief was measured by owners weekly before injections using the CBPI (canine brief pain inventory) validated scale. The 11 parameters of CBPI are grouped in three major domains: pain severity score (PSS), pain interference score (PIS) and overall impression of the quality of life (QoL).

**Results:**

Treatment with the p62-plasmid improved all 11 parameters of CBPI including PSS, PIS and QoL. Improvement in CBPI was observed after 2–4 weeks of treatment, whereas after 5–6 weeks of the treatment the parameters reached the plateau. After 10 weeks mean PSS score after the treatment decreased from 5.25 to 3.25, PIS score - from 7.0 to 3.27, and number of dogs with excellent and good QoL due to treatment increased from 1 to 12. Overall, the treatment success rate (i.e., a reduction ≥1 in PSS and ≥ 2 in PIS) was 90%. Importantly, no significant side effects of the p62-plasmid during the whole treatment period were observed.

**Discussion:**

In this pilot study Elenagen demonstrated efficacy in treatment of OA pain in dogs without side effects. The study has some limitations: small animal number, lack of long-term follow-up, and the outcome is limited to only one parameter, CBPI. Also in future studies the mechanism of anti-OA effect of p62 plasmid should be addressed.

## Introduction

Osteoarthritis (OA) is a progressive degenerative disease of synovial joints that characterized by structural and functional changes to the cartilage due to biomechanical and metabolic alterations (1). In humans, OA is the most common cause of disability worldwide among the elderly (2, 3). OA is also highly prevalent in dogs with about 20% of the canine population older than 1 year is affected by the disease (4). This results in lameness, loss of joint function and mobility, chronic pain, and reduced quality of life (5). Present in the knees of patients with OA, inflammation appears important contributor to disease pathogenesis. Pro-inflammatory cytokines such as IL-1, IL-6 and TNF are central to joint degeneration of OA, as well as to sensitization of pain neurons that innervate the joint capsule (30), although other cytokines, including IL-15, IL-17, IL-18, IL-21 are also implicated (31). These cytokines which produced by chondrocytes and synoviocytes (fibroblasts and macrophages), promote the cartilage-damaging activities of these cells (30) in particular via induction of metalloproteases (31). Pro-inflammatory cytokines, therefore, may be important targets for pharmaceutical intervention. There are three anti-inflammatory cytokines that are produced by the synovial membrane and cartilage, and are thus present in the synovial fluid of osteoarthritic joints: IL-4, IL-10 and IL-13. In addition, increased levels of IL-1Ra have been found which also has as an anti-inflammatory effect (8, 9). Other than surgical management for a select group of arthritic dogs, there are no disease-modifying therapies with strong evidence of efficacy in canine OA. Therefore, its management is mostly based on relieving the signs of the disease by treating pain and inflammation, and improving mobility and hence quality of life (10).

Non-steroidal anti-inflammatory drugs (NSAIDs) are still considered the cornerstone for the management of canine OA. However, in many cases, pain reduction is inadequate (6) and NSAID have deleterious effects when prescribed over long durations (7). They, in particular, can increase blood pressure, cause gastric ulcers, and even sometimes lead to acute kidney failure, stroke, or myocardial infarction (25, 31). Chronic systemic use of another common treatment modality, steroids, can lead to osteoporosis, aseptic joint necrosis, adrenal insufficiency, gastrointestinal, hepatic, and ophthalmologic effects (11). Among more recent treatments are non-steroidal, non-COX inhibiting drugs, the piprants (12, 13), anti-NGF (nerve growth factor) Mab Bedinvetma (14), mesenchymal stem cells (MSC) (15–17), but all of them also have limitation such as limited efficacy, short-term relief, and side effects (18).

Thus, although there is a number of treatments, OA-related pain management remains challenging and there is an urgent need for new effective and safe therapeutics for OA.

We have recently developed a biologic double-stranded supercoiled circular plasmid DNA coding for the protein SQSTM1/p62 (Elenagen). This protein plays multiple functions in the cells, in particular controlling autophagy, signal transduction, and inflammation (19, 20). Initially Elenagen was introduced as an anti-cancer vaccine. Indeed, Elenagen revealed anti-cancer activity in canine mammary tumors (21) as well as in clinical trials in humans and demonstrated a good safety profile (22–24). During our studies in animals, we found a quite unexpected effect of Elenagen which apparently unrelated to its vaccine action– it alleviated diseases of chronic inflammation (see ref. (19) for review) (22, 23, 29). In particular, we found that Elenagen decreases generation of pro-inflammatory cytokines such as TNF, IL-1, IL-6, increases anti-inflammatory cytokines (e.g., IL-4 and IL-10) and alleviates ovariectomy-induced osteoporosis in mice and rats (32, 33), Also, anti-inflammatory effect of Elenagen and decrease in disease symptoms were observed in rats with metabolic syndrome/obesity (34), and age-related macular degeneration (AMD) (35). Of note, all these inflammatory and anti-inflammatory cytokines are involved in OA as mentioned above (30, 31).

Given that OA is also considered as a disease of chronic inflammation, and canine OA is much closer to human OA than rodent models, we hypotheses that p62 plasmid may alleviate signs of OA. This pilot study was conducted to test this hypothesis in companion dogs.

## Materials and methods

This one-arm open label study of 17 companion dogs with OA was conducted in veterinary clinics in the Republic of Belarus in 2023–2024. All owners provided written confirmation of informed consent.

### Animal selection

Client-owned dogs weighing ≥ 3 kg of any age, sex and breed with a medical history, clinical signs, physical examination findings, and radiographic findings consistent with OA were recruited for inclusion in the study. Radiographic findings by radiologists included subchondral bone sclerosis, bone remodeling, osteophytes, irregular or diminished joint space at least at one joint. Only dogs with a diagnosis of OA made while screening for the study that had not commenced treatment of any type (including nutraceuticals, special diets, and over-the-counter supplement-type products) and dogs with a prior diagnosis of OA that the owners had elected not to treat were eligible for inclusion in the study. Animals were confirmed to be in good l health based on a general physical examination and routine blood (hematology and biochemistry) tests.

Dogs were excluded from the study if they have received:

(1) NSAIDs during the 2 weeks prior to evaluation for study enrolment.
(2) Glucocorticoids during the 4 weeks prior to evaluation.
(3) Opioids during the 4 weeks prior to evaluation.

Additional exclusion criteria:

(1) Any clinically important neurologic disease or orthopedic disease other than OA, as determined on the basis of history and results of a physical examination.
(2) Any chronic disease that required daily medication.
(3) A history of coagulopathy and unexplained bleeding episodes.
(4) Clinically important abnormalities detected on a complete blood count and serum biochemical testing at the time of the initial evaluation.

### Procedures and outcome measurement

The trial was a single-arm prospective interventional trial. The dogs were treated with the p62-plasmid, 1 mg intramuscular (IM) injection by a veterinarian once a week for 10 weeks, and the efficacy was assessed by the owners every week before injection using the Canine Brief Pain Inventory (CBPI). CBPI consists of three domains: the pain severity score (PSS, 0–10 scale), the pain interference score (PIS, 0–10) and the overall impression by an owner of the quality of life (QoL, “poor,” “fair,” “good,” “excellent”). To be eligible for the study, initial PSS > 2 and/or PIS > 2 were required.

The primary efficacy endpoint was treatment success at 10 weeks based on owner assessment of pain using CBPI. Treatment success was defined as a reduction ≥1 in PSS (0–10; 0 no pain, 10 extreme pain) and ≥ 2 in PIS (0–10; 0 no pain, 10 extreme pain) following the CBPI author recommendation (24, 26) compared with pre-treatment (baseline). Secondary efficacy endpoints included CBPI-based treatment success for all other assessed time points, the owner assessed PSS, PIS scores, and QoL and the percentage of dogs classified as a good’ or ‘excellent’ QoL at a final time point.

### Statistical methods

Quantitative data were described with medians and quartiles. Pre- and Post analysis of PSS, PIS and QoL was performed with a paired Mann–Whitney U test. Additionally in each subject and each score we quantified a trend of improvements via Spearman correlation *ρ* between the score value and time after the first intervention. Thus, in case of monotonic improvement/deterioration we would expect to see a *ρ* of −1 or +1, respectively. Afterward, a two-sided *t*-test was used to test the hypothesis that the expectation of *ρ* is 0. A threshold of 0.05 was chosen as a significance level, all tests were two-sided. Bonferroni-Holm adjustment was used for multiple testing corrections.

## Results

### Patient’s characteristics

Characteristics of the dogs enrolled in the study are presented in Table 1. As expected, most dogs with OA are Large breeds (e.g., 6 of 17 are Labradors), and of rather old age (median = 9 years), although smaller and younger dogs were also presented (e.g., 5-yr old Spitz, #9 Table 1). All dogs demonstrated chronic OA by radiographic finding and clinical signs, but were otherwise were in a good health.

**Table 1:** Patient characteristics.

#	Sex	Age	Weight, kg	Breed	Joints
1	f	11.5	35	Doberman	Stifle, lumbar spine
2	m	4.5	34	Akita	Stifle
3	m	8.5	6	Mixed	Hips (2), Stifle
4	m	6.5	50	Bernese Mountain Dog	Stifle, Hip
5	f	13.5	49	South Russian shepherd dog	Stifle
6	f	13	6	Mixed	Elbow (2), stifle
7	f	11	35	Labrador retriever	Elbow (2)
8	f	8	35	Labrador retriever	Elbow (2), stifle
9	m	5	3	Pomeranian Spitz	Elbow, stifle (2)
10	f	9.5	35	Labrador retriever	Elbow, stifle (2), tarsus (2)
11	f	11.5	32	German Shepherd	Hips, lumbar spine
12	f	11	35	Labrador retriever	Hips, stifle
13	m	14	40	Labrador retriever	Elbow
14	m	12	40	Labrador retriever	Elbow
15	m	6	3.2	YorkShire Terrier	Hips, stifle
16	f	6	9.5	Beagle	Hips, stifle
17	f	7.5	7.5	Fox Terrier	Hips

Median (range) 9 (4–14) 28 (3–50).

### Efficacy of treatment of p62 plasmid in dogs with OA

**Figure 1:**
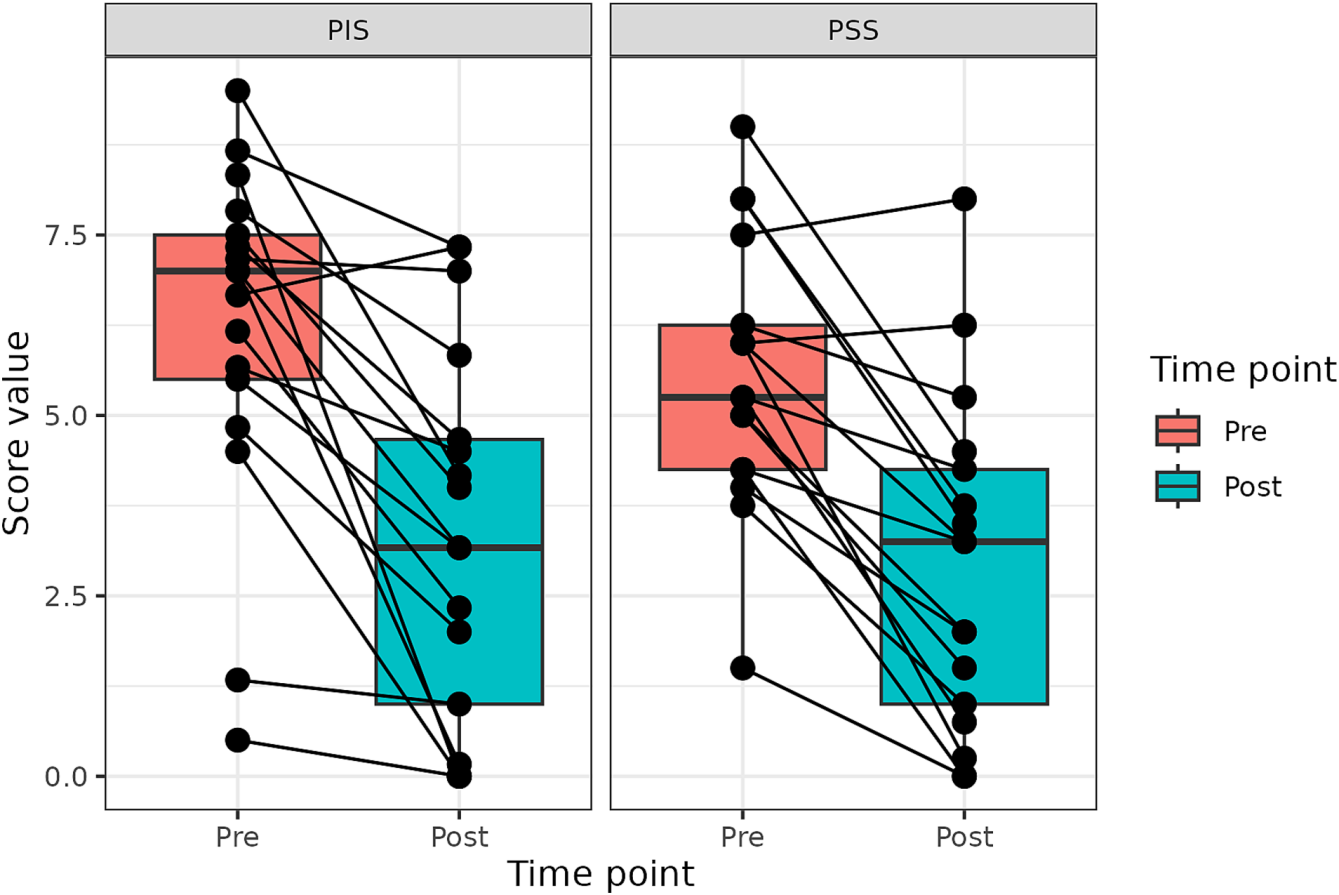
Changes in PIS and PSS scores after the treatment with p62 plasmid (described with a median, Q1 and Q3). Time points – before treatment start, and after 10 injections (10 weeks).

The dogs enrolled in the study were treated IM with 1 mg of the p62-plasmid once a week for 10 weeks. As in previous studies in dogs and humans, no adverse effects of the treatment including vomiting, diarrhea, colic, seizure were observed over the study period. For treatment effect a standard CBPI test which was done by dog owners was used every week before injections. The changes in main components of CBPI, the pain severity score (PSS), pain interference score (PIS), and quality of life (QoL) in individual dogs after treatment are presented in Table 2 and Figure 1.

**Table 2:** Efficacy of p62 plasmid treatment in dogs with OA.

Dog ID	Time point	PSS	PIS	QoL
1	Pre	6.00	7.00	Fair (1)
	Post	0.25	0.17	Good (2)
2	Pre	8.00	7.50	Good (2)
	Post	3.75	4.00	Good (2)
3	Pre	4.25	8.33	Fair (1)
	Post	0.00	0.00	Excellent (3)
4	Pre	3.75	1.33	Fair (1)
	Post	1.00	1.00	Good (2)
5	Pre	6.25	7.83	Fair (1)
	Post	5.25	5.83	Fair (1)
6	Pre	4.25	7.00	Fair (1)
	Post	3.25	3.17	Fair (1)
7	Pre	5.00	4.83	Fair (1)
	Post	1.50	2.00	Good (2)
8	Pre	1.50	4.50	Fair (1)
	Post	0.00	0.00	Excellent (3)
9	Pre	5.00	7.17	Fair (1)
	Post	2.00	7.00	Poor (0)
10	Pre	6.00	7.33	Poor (0)
	Post	3.25	4.67	Good (2)
11	Pre	4.00	6.17	Fair (1)
	Post	2.00	2.33	Good (2)
12	Pre	7.50	8.67	Fair (1)
	Post	8.00	7.33	Fair (1)
13	Pre	6.00	6.67	Fair (1)
	Post	6.25	7.33	Fair (1)
14	Pre	9.00	9.50	Fair (1)
	Post	4.50	4.17	Good (2)
15	Pre	5.25	0.50	Fair (1)
	Post	0.75	0.00	Excellent (3)
16	Pre	8.00	5.50	Fair (1)
	Post	3.50	3.17	Good (2)
17	Pre	5.25	5.67	Fair (1)
	Post	4.25	4.50	Good (2)

Time points – pre – before treatment, post – after 10 injections (10 weeks).

Based on CBPI assessment, only one dog (#13) demonstrated some worsening of CBPI scores, although less than 1 unit in value, and this dog was the oldest one in the population (14 year old Labrador retriever) (Table 1). Another dog, #9, show worsening of QoL (from fair to poor), and this was the smallest dog (Pomeranian Spitz). All other dogs demonstrated a varying degree of improvement in all three components of CBPI score, with some of them (#3, #8) reporting disappearance of all signs of OA by the end of the treatment (Table 1). Thus, as whole, the treatment with the p62-plasmid was effective in 15 of 17 dogs (90%), i.e., there was reduction ≥1 in PSS and ≥ 2 in PIS (36, 37) compared with pre-treatment (baseline).

Change in PSS and PIS scores for all dogs is presented in Figure 1. Both PSS and PIS scores decreased significantly by the end of the treatment. Specifically, average PSS score decreased from 5.25 to 3.25 (*p* < 0.001) and average PIS score from 7.0 to 3.17, (*p* < 0.001). Additionally, a significant improvement in QoL was observed: only 6% of dogs had good QoL before treatment, but after the treatment good QoL was observed in 53% of dogs (Figure 1).

Besides assessment by CBPI, short video clips of dog’s behavior were made before and after the study. These clips also demonstrate improvement in dog’s mobility after the treatment (see Supplementary material).

**Figure 2:**
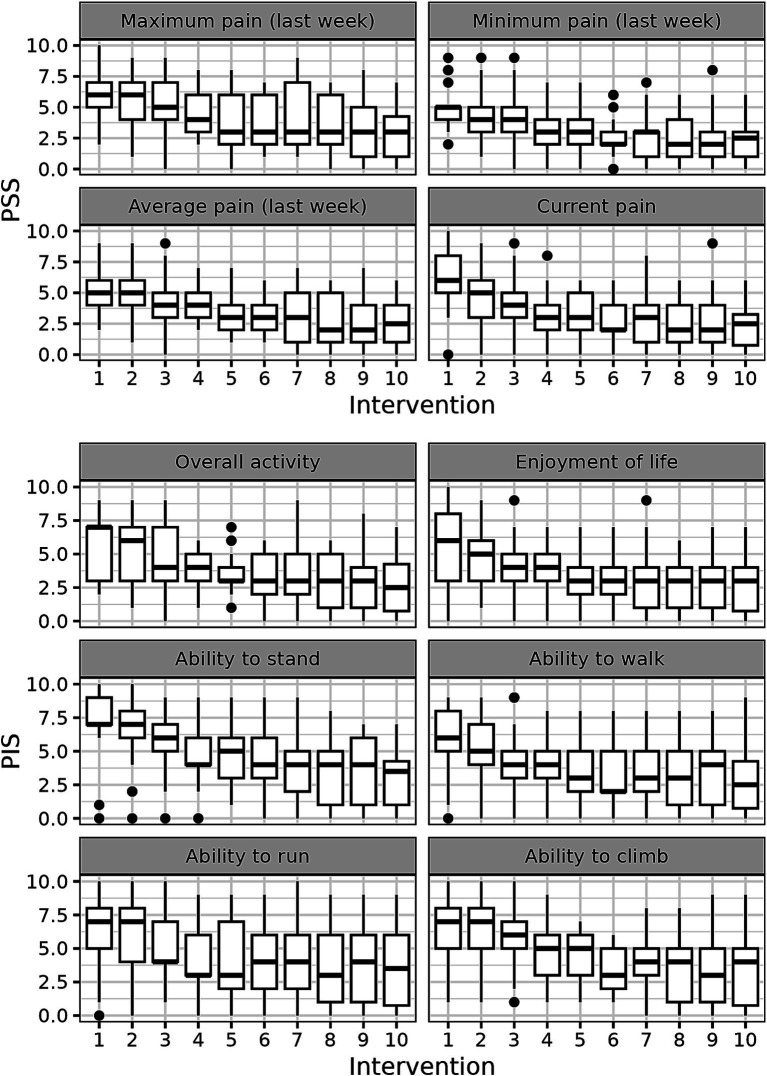
CBPI score dynamics during the treatment with p62 plasmid 10 quantitate parameters of CBPI scores were measured in each dog every week and their medians are presented.

We next addressed the question how each of the 10 components of CBPI score changed before and during treatment in the whole dog population. The positive effect of the treatment started to be observed mostly after 2–4 weeks of treatment, whereas after 5–6 weeks of the treatment the parameters reached the plateau (Figure 2).

**Figure 3:**
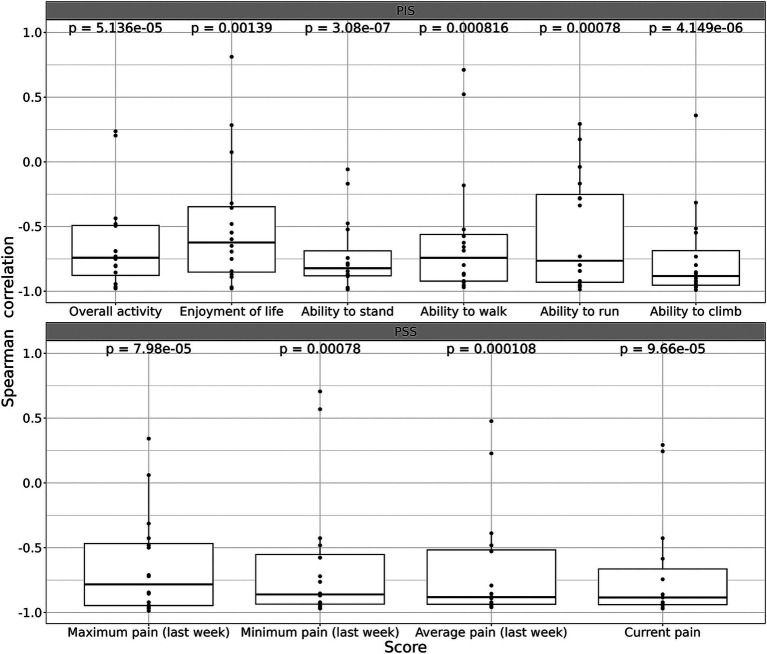
Spearman correlation *ρ* for each parameter of subjective assessment (CBPI score). See Materials and Methods for details. Trend of improvements was quantified a via Spearman correlation *ρ* between the score value and time after the first intervention. In case of monotonic improvement/deterioration, *ρ* of −1 or +1 is expected, respectively. Afterward, a two-sided *t*-test was used to test the hypothesis that the expectation of *ρ* is 0. A threshold of 0.05 was chosen as a significance level, all tests were two-sided. Bonferroni-Holm adjustment was used for multiple testing corrections.

To understand the relationship between the duration of injections time and the behavioral scores, calculated the Spearman correlation coefficients was calculated. We graph the Spearman correlation coefficients for each subjective assessment in Figure 3. The calculations show that for all 10 parameters of CBPI scores there is a clear inverse correlation between scores and the time of the treatment, with several parameters (e., pain levels) close to −1.0 (Figure 3). Overall, correlation of all parameters with time of treatment demonstrates statistical significance ranging from 4×10^−6^ to 0.0014 (Figure 3).

## Discussion

OA is regarded as a significant cause of pain, lameness and morbidity in humans, dogs and some other animals (10, 27).

Here we present the results of our pilot clinical study of a novel biologic, a DNA plasmid encoding SQSTM/p62, in companion dogs suffering from OA. The dogs in the study were injected IM with the p62-plasmid once a week for 10 weeks. As the main criterion for clinical efficacy we employed a standard CBPI questionnaire that was provided weekly to the owners. CBPI is widely used in OA studies in dogs and includes 11 parameters constituting three major domains: the pain severity score (PSS) the pain interference score (PIS) and the overall impression of the quality of life (QoL) (24, 26).

We found that the treatment with the p62-plasmid significantly improved all 11 parameters of CBPI as well as PSS, PIS and QoL (Figure 1, Tables 2, 3). Specifically, mean PSS score after the treatment decreased from 5.25 to 3.25 (i.e., 1.6 times), PIS score - from 7.0 to 3.27 (2.2 times), and number of dogs with excellent and good QoL due to treatment increased from 1 to 12 (Table 2). Overall, the treatment success rate (i.e., a reduction ≥1 in PSS and ≥ 2 in PIS) was 90%. Importantly, similar to our previous studies with dogs and humans, no side effects of the p62-plasmid during the whole treatment period were observed.

**Table 3:** Change in quality of life (QoL) of dogs after the treatment with p62 plasmid for 10 weeks, *n* (%).

QoL	Before	After
Poor (0)	1 (5.9%)	1 (5.9%)
Fair (1)	15 (88%)	4 (24%)
Good (2)	1 (5.9%)	9 (53%)
Excellent (3)	0 (0%)	3 (18%)

QoL = *p* < 0.001.

Time points –before treatment start, and after 10 injections (10 weeks).

Analyzing the time course of changes in CBPI scores during the treatment, we observed that all parameters of CBPI significantly decreased with time although with somewhat different dynamics (Figures 2, 3). The scores already began to improve after 3–4 injections (i.e., after 3–4 weeks), and reached the plateau after approximately 5–6 injections (Figures 2, 3).

There is currently no cure for OA and most of the treatments are basically symptomatic in order to manage pain, stiffness and swelling (17, 18, 28). In other words, the goal of available therapies is to delay the progression of the disease, reduce pain and restore mobility, with the final objective to improve the overall quality of life (18, 36).

We compared the results of our study with some previous trials of other anti-OA drugs in dogs. For instance, in dogs receiving NSAID drug carprofen, there was decrease in median PSS scores from 4.25 to 2.25, and PIS score from 4.33 to 2.67 on days 0 and 14, respectively (24). In the trial of currently approved anti-NGF monoclonal Ab (Librela, Zoetis), decrease in PSS score was from 4.1 to 3.0, and PIS score – from 4.5 to 3.2 after 6 weeks of treatment with subsequent plateau till 12 weeks (end of the trial); the treatment success rate which was assessed as described above being 57% comparing to 34% in placebo group (14). Thus, comparing to these other currently used medications mentioned above, efficacy of the p62-plasmid for treatment of OA looks very promising.

There are several limitations of the study. First limitation of the study is a small number of dogs; however despite this, the results are highly statistically significant. The second limitation of the study is the absence of a control (placebo) group. It should be noted that using a placebo as a control in a disease that is known to be progressing painful and for which there are some treatments available has ethical implications (18).

Thus, in future we plan to conduct randomized two-arm study with our plasmid and another approved medication (e.g., Librela) for comparison. Other limitations are using only one parameter, CBPI, and the absence of long term follow-up.

During previous studies, we made an unexpected observation that the p62-plasmid, besides anticancer activity, can alleviate osteoporosis and inflammation via anti-inflammatory cytokines and chemokine production. Among the pro-inflammatory cytokines suppressed by the p62-plasmid are IL-1, IL-6, TNF, while anti-inflammatory cytokines IL-4, IL-10 and IL-1RA were increased (32, 33). Since chronic inflammation is currently recognized as important contributor in pathogenesis of OA, in particular, in OA-associated pain (30), we hypothesed that p62 plasmid may alleviate signs of OA via anti-inflammatory mechanisms found in our previous studies in osteoporosis and metabolic syndrome (32–34).

Although the mechanism of the p62-plasmid action in OA is out the scope of this study, we believe that anti-inflammatory effect may play an important role. Indeed, the effect of p62 plasmid began to observed already after 4 weeks, and although no any radiographic improvements of OA were found up to 10 weeks (data not shown). On the other hand clear pain alleviation is seen in treated dogs, which may indicate anti-inflammatory effect of p62 plasmid since OA pain in particular, caused by inflammation (30) Further studies of the p62-plasmid are needed to measure inflammation biomarkers and assess a proposed mechanism of action of the p62-plasmid in OA. Also, the present study may indicate potential applications of the p62-plasmid as anti-pain medication in other diseases associated with a chronic inflammation.

## Data Availability

The raw data supporting the conclusions of this article will be made available by the authors, without undue reservation.

## Supplementary material

SUPPLEMENTARY VIDEOS 1–3 Video clips of dog’s behavior before and after completion of the study. These clips also demonstrate improvement in dog’s mobility after the treatment.
